# Exudative Retinal Detachment After ROP Laser Photocoagulation

**DOI:** 10.7759/cureus.27891

**Published:** 2022-08-11

**Authors:** Henry Zou, Lauren Fletcher-Morehouse

**Affiliations:** 1 Ophthalmology, Michigan State University College of Human Medicine, Grand Rapids, USA; 2 Ophthalmology, Helen DeVos Children's Hospital, Grand Rapids, USA

**Keywords:** neurosensory retina, retinal pigment epithelium, laser photocoagulation, exudative retinal detachment, retinopathy of prematurity

## Abstract

Exudative retinal detachment (ERD) can be a rare postoperative complication of laser photocoagulation surgery when used to treat type I retinopathy of prematurity (ROP). We present a case of bilateral ERD following ROP laser photocoagulation. A preterm male infant born at 24 weeks gestation and weighing 600 grams was diagnosed with stage 3/zone II/pre-plus ROP in both eyes. He was on oxygen therapy for 92 days due to chronic lung disease and was treated with laser photocoagulation at 40 weeks postmenstrual age. Initial laser settings (in use for over 15 years) were 300 mW power, 300 ms duration, and 300 ms intervals. Due to strong laser absorption, power was decreased to 250 mW for most of the procedure. He was prescribed prednisolone acetate drops four times per day for postoperative care. One week later, he developed complete ERD in both eyes. The patient was monitored in the neonatal intensive care unit (NICU) for three weeks and prednisolone acetate drops were increased to every two hours and tapered over one month. Complete resolution of ERD with residual peripheral exudate bilaterally was observed eight weeks after surgery.

This case suggests that even after the settings of an ROP laser have been used safely for 15 years, it is important to tailor settings for each individual patient utilizing the least power and duration for laser application as possible. Furthermore, this case highlights the importance of titrating laser power in response to spot blanching throughout the procedure. However, near-complete resolution of post-ROP laser ERD is possible with minimal changes to standard postoperative management.

## Introduction

Retinopathy of Prematurity (ROP) is a disease that involves the incomplete growth of blood vessels in the retina followed by the abnormal proliferation of blood vessels into the vitreous and is the leading cause of preventable childhood blindness in the United States [1,2]. Surgical intervention is indicated for severe pre-threshold ROP, termed type I ROP, and argon or diode laser photocoagulation is considered the gold standard of treatment [3,4]. Exudative retinal detachment (ERD) is a rare postoperative complication [2,5,6]. Literature exists documenting two cases of ERD seen as the initial presenting sign in ROP [7]. We present a case of bilateral ERD following laser photocoagulation in an ROP patient.

This paper was presented at the 2022 Annual Clinical Assembly of the American Osteopathic Colleges of Ophthalmology and Otolaryngology-Head and Neck Surgery, May 11 to 14, 2022.

## Case presentation

**Figure 1:**
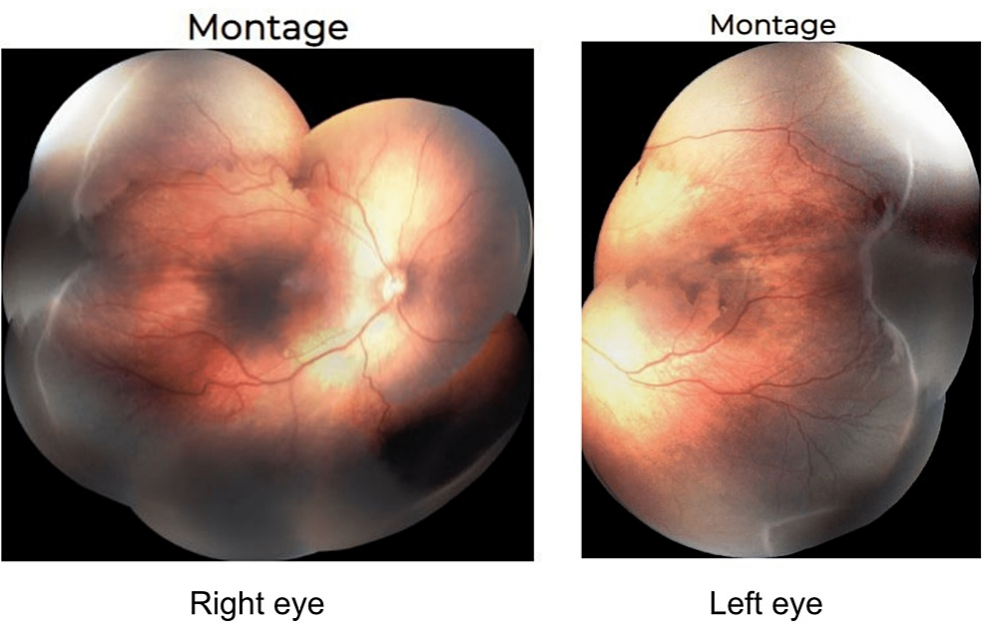
Pre-operative montages of both eyes

A 600 gm premature African-American male infant was born at 24 weeks gestational age. His past medical history included anemia, chronic lung disease, bronchopulmonary dysplasia, patent ductus arteriosus, patent foramen ovale, anemia, and feeding difficulties. He underwent 92 days of supplemental oxygen therapy for his chronic lung disease, which involved 21% oxygen delivered via a high-flow nasal cannula. Examination at 40 weeks postmenstrual age (PMA) indicated stage 3/zone II/pre-plus ROP in both eyes (Figure 1). During the examination, fibrotic ridges and significant subretinal fluid throughout the avascular retina were seen in both eyes. Neither rubeosis nor vitreous haze was seen in either eye. Despite not meeting the threshold for type I ROP, it was determined that laser photocoagulation was indicated given the significant subretinal fluid in both eyes. A red diode 810 nm Oculight SLx laser (IRIDEX, Mountain View, CA, USA) was used to treat both eyes three days after the exam; power was initially set to 300 mW for a 300 ms duration at 300 ms intervals, and 850 to 950 spots were administered to each eye. Due to strong laser absorption at 300 mW power, power was immediately decreased to 250 mW for the duration of the procedure, increasing to 300 mW only superiorly at clock hours 11 to 12 and one to two in both eyes where the laser would not take up well. No perioperative complications were noted, and the patient was prescribed prednisolone acetate drops four times per day for postoperative care. 

**Figure 2:**
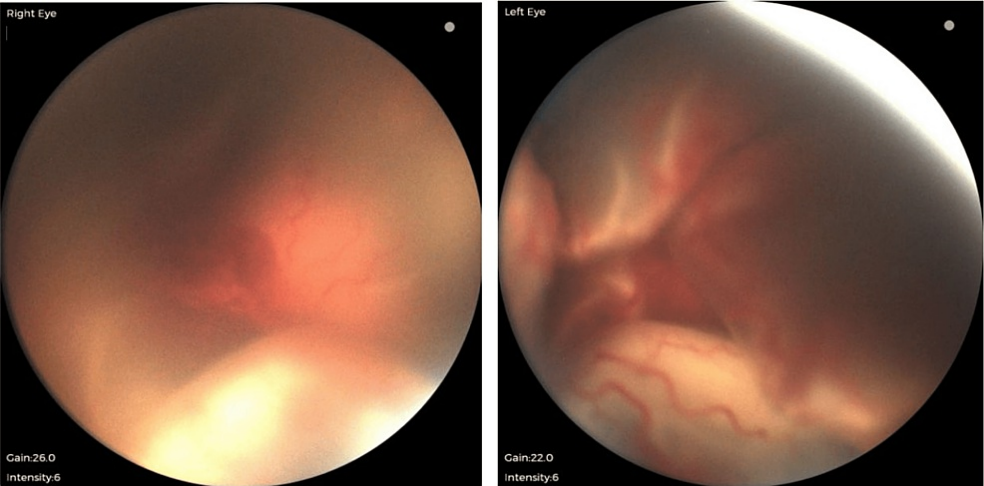
Exudative retinal detachment (ERD) of both eyes at one-week post-op

**Figure 3:**
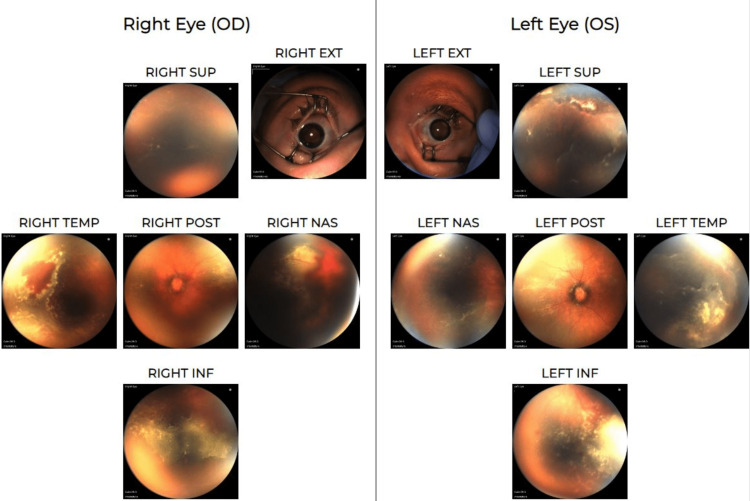
Near-complete ERD resolution in both eyes at four weeks post-op ERD: Exudative retinal detachment, OD: Right eye, OS: Left eye, EXT: Exterior view, SUP: Superior view, POST: Posterior view, NAS: Nasal view, TEMP: Temporal view, INF: Inferior view

One week after laser photocoagulation, the patient presented with complete ERD in both eyes (Figure 2). Based on the recommendations of a pediatric vitreoretinal specialist, topical prednisolone acetate drops were increased to every two hours and tapered over one month. The patient was subsequently monitored for the next three weeks in the NICU, during which most of the subretinal exudate appeared to have been resorbed (Figure 3). During a follow-up outpatient visit 8 weeks after surgery (48 weeks PMA), the patient was observed to have complete resolution of ERD with residual peripheral exudate in both eyes. At the 11-month follow-up visit, a comprehensive visual exam yielded CSUM (central, steady, unmaintained fixation) at distance and near in the right eye, and CSM (central, steady, maintained fixation) at distance and near in the left eye via the induced tropia test, grossly full bilateral visual fields, and a right esotropia via the Krimsky test. The fundus exam revealed a blunted reflex with scarring in the macula and a chorioretinal scar temporal to the optic nerve in the right eye; peripheral 360° laser scars were visualized in both eyes. Patching therapy was initiated.

## Discussion

Overtreatment due to the laser power setting being too high, treatment duration being too long, or failure to reduce power when moving from the posterior retina to the thinner anterior retina can induce iatrogenic retinal tears and detachment [8,9]. However, there does not appear to be an internationally recognized standard on the safest or most efficacious range of initial power or duration settings. Jalali et al. from India recommended starting with 250 mW power for 150 ms duration, and Ells et al. from Canada recommended starting with 200 mW for 200 ms for stage 3/zone II ROP cases [4,8]. Houston et al. from the USA described a range of initial settings among institutions from 200 to 400 mW power for 100 to 300 ms duration [9]. Retinal pigmentation and intended areas of treatment are considerations when selecting initial laser settings. However, there is a lack of definitive or standardized guidelines on the influence of these factors [4]. The initial settings of 300 mW power for 300 ms duration at 300 ms intervals, in this case, had been used successfully with this institution’s particular laser on ROP patients for the past 15 years. In a similar case of post-laser ERD in a patient with type 1 ROP (stage 3/zone II/plus) at the University of Michigan, the settings ranged from 200 to 300 mW for 100 to 200 ms duration [6]. Nonetheless, this case highlights the importance of tailoring laser settings for individual patients, using the minimum necessary power and duration, and always titrating the laser energy by spot blanching throughout the procedure. 

Another hypothesis for post-ROP laser ERD is that the laser damaged the retinal pigment epithelium (RPE) and choriocapillaris, which subsequently induced damage to the blood-retinal barrier and impaired the shunting of fluid from the retinal periphery to the posterior pole [5]. This phenomenon was demonstrated in cats [5]. It is hypothesized that a PMA of 40+ weeks at treatment is a risk factor as a more mature choriocapillaris could exacerbate the impairment of fluid shunting to the posterior pole [4]. However, this is disputed by multiple cases of post-ROP laser ERD seen at 37 weeks PMA or less [5]. Additional possible risk factors include low birth weight, multiple gestations, pulmonary hypertension, and neonatal sildenafil administration [6]. In this case, the patient underwent laser photocoagulation at 40 weeks PMA, the preoperative exam identified subretinal fluid throughout the avascular retinas of both eyes, and the patient’s mother had multiple gestations, which may have predisposed him to experience postoperative ERD.

Given the rarity of post-ROP laser ERD, there is currently no consensus on care management [6]. Two cases that affected only the macula and were diagnosed immediately after surgery reportedly self-resolved without sequelae in two to three weeks [6]. Three cases were treated with intravenous dexamethasone and daily topical prednisolone acetate 1%; all three experienced either complete or near-complete resolution of ERD four to nine weeks after surgery [2]. A case in the right eye was treated with tobramycin and dexamethasone ointment four times daily; the resolution was seen four weeks after surgery, but there were residual subretinal exudates and outer retinal atrophy [5]. A case in the left eye was treated with intravenous dexamethasone every 12 hours tapered over 10 days and topical prednisolone acetate 1% every two hours; near-complete resolution occurred three weeks after surgery [6]. However, multifocal subretinal lipid deposits were seen at 62 weeks of PMA [6]. Our patient experienced near-complete resolution of post-ROP laser ERD with minimal changes to postoperative care.

## Conclusions

Although this institution's standard laser settings have been used safely for over 15 years, it is important to recognize the importance of tailoring these settings for each individual patient utilizing the least power and duration of laser application possible. Moreover, this case illustrates the need to titrate laser energy in response to spot blanching throughout the procedure to reduce the risk of injury and postoperative sequelae. The risk of inflammation and subsequent exudative retinal detachment should be included in pre-operative discussions regarding risks and complications. Furthermore, this case suggests that near-complete resolution of post-ROP laser ERD is possible with minimal changes to standard postoperative care. Nonetheless, the lack of evidence-based consensus on the etiology, risk factors, and treatment of post-ROP laser ERD highlights the need for more research on this complication. Future studies should strive to standardize the ranges of safe and efficacious power, duration, and interval settings of ROP lasers, identify definitive risk factors, and ascertain optimal management guidelines. 
